# Repair Bond Strength of Conventionally and Digitally Fabricated Denture Base Resins to Auto-Polymerized Acrylic Resin: Surface Treatment Effects In Vitro

**DOI:** 10.3390/ma15249062

**Published:** 2022-12-19

**Authors:** Mohammed M. Gad, Zainab Albazroun, Fatimah Aldajani, Ahmed M. Elakel, Mai El Zayat, Sultan Akhtar, Soban Q. Khan, Saqib Ali, Ahmed M. Rahoma

**Affiliations:** 1 Department of Substitutive Dental Sciences, College of Dentistry, Imam Abdulrahman Bin Faisal University, P.O. Box 1982, Dammam 31441, Saudi Arabia; 2 College of Dentistry, Imam Abdulrahman Bin Faisal University, P.O. Box 1982, Dammam 31441, Saudi Arabia; 3 Department of Preventive Dental Sciences, College of Dentistry, Imam Abdulrahman Bin Faisal University, P.O. Box 1982, Dammam 31441, Saudi Arabia; 4 Department of Biophysics, Institute for Research and Medical Consultations (IRMC), Imam Abdulrahman Bin Faisal University, P.O. Box 1982, Dammam 31441, Saudi Arabia; 5 Department of Dental Education, College of Dentistry, Imam Abdulrahman Bin Faisal University, P.O. Box 1982, Dammam 31441, Saudi Arabia; 6 Department of Biomedical Dental Sciences, College of Dentistry, Imam Abdulrahman Bin Faisal University, P.O. Box 1982, Dammam 31441, Saudi Arabia; 7 Department of Restorative Dental Sciences, College of Dentistry, Imam Abdulrahman Bin Faisal University, P.O. Box 1982, Dammam 31441, Saudi Arabia

**Keywords:** CAD-CAM, bond strength, denture repair, surface treatment

## Abstract

Denture base fracture is one of the most annoying problems for both prosthodontists and patients. Denture repair is considered to be an appropriate solution rather than fabricating a new denture. Digital denture fabrication is widely spreading nowadays. However, the repair strength of CAD-CAM milled and 3D-printed resins is lacking. This study aimed to evaluate the effect of surface treatment on the shear bond strength (SBS) of conventionally and digitally fabricated denture base resins. One l heat-polymerized (Major base20), two milled (IvoCad, AvaDent), and three 3D-printed (ASIGA, NextDent, FormLabs) denture base resins were used to fabricate 10 × 10 × 3.3 acrylic specimens (N = 180, 30/resin, *n* = 10). Specimens were divided into three groups according to surface treatment; no treatment (control), monomer application (MMA), or sandblasting (SB) surface treatments were performed. Repair resin was bonded to the resin surface followed by thermocycling (5000 cycles). SBS was tested using a universal testing machine where a load was applied at the resin interface (0.5 mm/min). Data were collected and analyzed using ANOVA and a post hoc Tukey test (α = 0.05). SEM was used for failure type and topography of fractured surfaces analysis. The heat-polymerized and CAD-CAM milled groups showed close SBS values without significance (*p* > 0.05), while the 3D-printed resin groups showed a significant decrease in SBS (*p* < 0.0001). SBS increased significantly with monomer application (*p* < 0.0001) except for the ASIGA and NextDent groups, which showed no significant difference compared to the control groups (*p* > 0.05). All materials with SB surface treatment showed a significant increase in SBS when compared with the controls and MMA application (*p* < 0.0001). Adhesive failure type was observed in the control groups, which dramatically changed to cohesive or mixed in groups with surface treatment. The SBS of 3D-printed resin was decreased when compared with the conventional and CAD-CAM milled resin. Regardless of the material type, SB and MMA applications increased the SBS of the repaired resin and SB showed high performance.

## 1. Introduction

Digital denture fabrication is a modern technology aiming to overcome the drawbacks of conventionally fabricated poly methyl methacrylate (PMMA) [1]. Two CAD-CAM methods were reported, namely the subtractive manufacturing (SM, milled) and additive manufacturing (AM, 3D-printed) methods [1,2]. Each technique aims to create a prosthesis with high mucosal adaptability, providing accurate retention, stability, and support with minor fabrication distortion. In the SM method, the denture is milled for pre-polymerized acrylic discs by using milling burs to the designed configurations, while in AM the technology consists of building an object layer by layer using photopolymerized fluid resins with different printing technologies and printing parameters [3,4,5].

In the SM method, PMMA blocks are manufactured and polymerized under high temperatures and pressure, which is supposed to produce highly condensed and low-porosity materials with chemical and mechanical properties that are higher than those of conventionally processed PMMA [6]. Additionally, SM dentures are clinically implemented with more accuracy and adaptation and patient satisfaction when compared with conventional PMMA [7]. The AM method retains accuracy and has faster production cycles than conventional manufacturing, which improves the patient’s remedy upon wearing the denture [8,9]. Additionally, AM has a lower cost due to no waste materials, no deterioration of milling burs, and multiple productions of designed materials with high accuracy. However, AM still has some limitations for clinical applications due to low mechanical performances in comparison to SM and conventional methods [8].

Considering both methods’ implementations for denture fabrication, wide distributions, and advancement of digital technologies, the denture may be fractured due to flexing action and different intraoral stresses or impact forces [10,11]. The most frequent reasons for denture fracture have been found to be unintended fall, poor fit, bad occlusion, and other reasons related to material failure [12]. Instead of a new denture fabrication, repair is one of the most acceptable solutions in the case that fractured parts can easily restore the denture’s original relation. Many factors affect the repair strength, such as repair material type, repair surface design, and repair surface treatment [13]. Chemical and mechanical surface treatments were suggested, and increased bond strength was reported by both surface treatments in comparison to untreated groups [14,15].

Monomer application showed an increase in the bond strength of repaired resin [16]. The basic mechanism is that MMA can dissolve the surface of PMMA, which creates more growing of extra superficial pits and cracks, thus increasing the surface area that is beneficial for bonding [17,18]. One of the mechanical surface treatments is the air-abrasive particles, which causes roughening of the surface with different sizes of alumina particles [19]. The alumina abrasive particles result in surface changes that increase the surface bonding area and subsequently improve the repair bond strength [14]. Additionally, when compared with the MMA application, alumina blasting showed better performances alone or when combined with bonding agents [15].

Repair strength can be evaluated by measuring the flexural strength by fabricating bar specimens. This method is reliable to measure both the strength of the repaired resin and the resin–repair interface [14]. Both shear bond strength and tensile bond strength are methods of evaluating the bond at the resin–repair interface [20]. In shear bond strength (SBS), a load was applied at the interface exerting vertical forces, aiming to debond the repaired resin [20]. Hence, SBS was selected for evaluating the repair bond strength focusing on the effect of surface treatments with different denture base resins.

Although multiple studies were carried out on both technologies for denture base fabrications, limited studies were performed to assess the repairability of 3D-printed resins. Moreover, there is a lack of comparison between the repair strength of CAD-CAM milled, 3D-printed, and conventional PMMA denture base resins. Our study was carried out to compare the repair bond strength of milled, 3D-printed, and conventional PMMA resins with different surface treatments. The null hypothesis was that there is no significant difference in the repair bond strength between the tested denture base resins and repair resin as well as the surface treatment effect.

## 2. Materials and Methods

### 2.1. Specimens Preparation

Sample size calculation was performed through power analysis by using the online calculator “Power and Sample size.com” (http://powerandsamplesize.com/Calculators/, accessed on 13 August 2022). The method of comparison of two means was selected for the sample size calculation. Hence, averages and standard deviation were used along with the power, which was set as 80%, and the level of significance was set as 0.05. Hence, the calculated sample size was 10 for each group. The denture base materials used in this study and the method of fabrication are summarized in Table 1. Acrylic specimens (N = 180, 30/resin, n = 10) were prepared in the dimensions 10 × 10 × 3 mm according to manufacturer’s recommendations. All of the specimens were standardized regarding the polishing method and one investigator. The prepared specimens were polished using silicon carbide abrasive papers with different grits (500, 800, 1200) that mounted on an automated polishing machine (Metaserve 250 grinder-polisher; Buehler, Lake Bluff, IL, USA).

**Figure 1:**
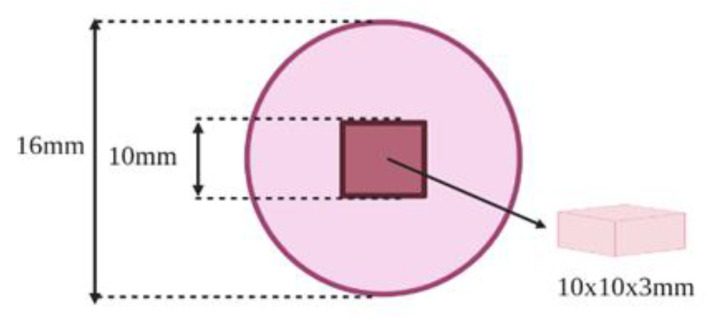
Illustration of the specimen (10 × 10 × 3 mm) centered in the center of resin holder (16 × 10 mm).

One resin holder (16 × 10 mm) per specimen was fabricated using a silicon mold. Each specimen was placed and centered at the bottom of a silicon jig and then the mixed autopolymerized resin was poured to fill the jig. After polymerization, the resin flashes were removed and we confirmed that the resin specimen dimensions are clear (Figure 1).

### 2.2. Surface Treatment

Each acrylic resin base was further divided into three groups (n = 10) according to the surface treatment; the first group was without surface treatment (control); the second group was treated with monomer applications by using a microbrush in one direction for 180 s (MMA); and the third group was air-abraded with 50 μm aluminum oxide particles (SB) using a sandblasting machine (Wassermann Dental Machine, GmbH, Hamburg, Germany) from 1 cm distance for 10 s with 2.5 bar pressure. A customized jig with an inner diameter of 6 mm was used to determine the area of sandblasting application.

### 2.3. Repair Procedures

**Figure 2:**
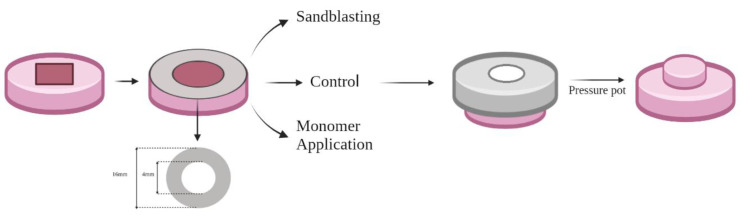
Illustration of the specimen surface treatment and repair procedures’ standardization.

A plastic cylinder (4 mm diameter × 6 mm length) was fixed on the center of the prepared acrylic resin specimen within the holder. A customized jig was prepared for all specimens’ repair. Each specimen with surface treatment was fixed in the jig and then the autopolymerized repair resin (Major repair, Major Prodotti Dentari SPA, Moncalieri, Italy) was mixed and packed in the space fabricated by a plastic cylinder (Figure 2). The mold with a repaired specimen was overfilled and covered with a glass slap under pressure (1 Kg) for 10 min and then placed into a pressure pot (2 bar pressure at 40 °C) for 15 min [13,21]. After polymerization, the plastic cylinder was removed followed by the specimens’ storage in distilled water (37 °C) for two days; they were then subjected to thermocycling for 5000 cycles, at 5 °C to 55 °C with a 30 s dwell time within a thermocycling machine (Thermocycler, THE-1100/THE-1200, SD Mechatronik GMBH Miesbacher, Westerham Germany).

### 2.4. SBS Testing

Shear bond strength measurement was performed with a universal testing machine (Instron, Instron Corp. Norwood, MA, USA). Each specimen was fixed to the customized machine jig with a uni-beveled chisel and the load was applied as close as possible at the resin interface with cross head speed (0.5 mm/min) until specimen failure. The SBS was calculated using the equation SBS (MPa) = F/A, where F (N) and A are the bonding area. After fracture, the specimens were analyzed concerning the nature of failure (adhesive, cohesive, mixed) using an optical microscope (Nikon, H550L, Tokyo, Japan) at 10-fold magnifications followed by specimens’ selection per group for further analysis using scanning electron microscopy (SEM).

### 2.5. Scanning Electron Microscope (SEM) Analysis

**Figure 3:**
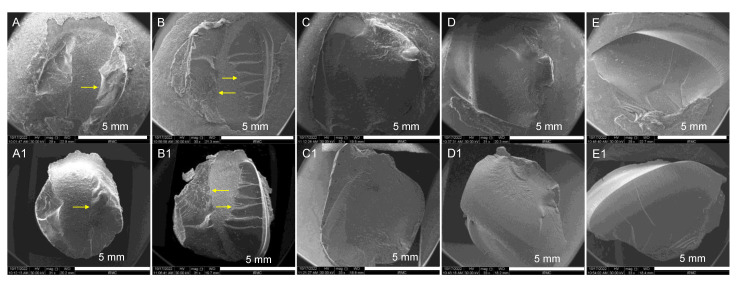
Nature of failure results of 3D-printed resins. Representative SEM images of (**A**–**E**) denture base resins side and (**A1**–**E1**) repair resin side: (**A**,**A1**) IvoCad, (**B**,**B1**) NextDent, (**C**,**C1**) FormLabs, (**D**,**D1**) AvaDent, and (**E**,**E1**) ASIGA; arrows indicate mixed failure (**A**,**B**), and cohesive failure (**C–E**) within the 3D-printed resin surface. SEM magnifications: ×29–×33, scale bars = 5 mm.

SEM (FEI, Inspect S50, Czech Republic) was applied to analyze the nature of fracture (i.e., adhesive, cohesive, or mixed) for the represented specimens (IvoCad, NextDent, FormLabs, AvaDent, and ASIGA). For evaluating the complete behavior of fractures, both parts of the fracture sides (denture base resins side and repair resin side) were mounted onto SEM stubs and examined under SEM after gold coating. SEM was operated at an accelerating voltage of 30 kV to obtain SE images. Low magnification images were taken to inspect the full view of the fractures for each specimen and are displayed in Figure 3 in the magnification range of ×29–33.

### 2.6. Statistical Analysis

Statistical package for social sciences (SPSS v.23) was used for data analysis. Averages and standard deviations were calculated as a part of the descriptive analysis of the data. The normality of the data was tested by using the Shapiro–Wilk test and insignificant *p*-values from the test showed that the data were normally distributed. Hence, parametric tests were used for inferential data analysis. One-way ANOVA was used to test the effect of surface treatment or type of material on the tested property followed by the Tukey (HSD) post hoc test. Two-way ANOVA was used to study the combined effect of surface treatment and materials on the tested property. All *p*-values smaller than 0.05 were considered statistically significant.

## 3. Results

Based on one-way ANOVA analysis, the overall variation was found to be statistically significant (*p* < 0.0001) and hence a post hoc test was performed. Table 2 shows the SBS comparison per material concerning the surface treatment effect (horizontally). Comparing materials without surface treatment, no significant differences in SBS between HP, IvoCad, and AvaDent groups were found (*p* > 0.05). By contrast, the 3D-printed resins group showed a significant decrease in SBS (*p* < 0.0001) when compared with the HP, IvoCad, and AvaDent groups. For the 3D-printed groups without surface treatment, no significant differences were found between ASIGA, NextDent, and FormLabs (*p* > 0.05).

SBS increased significantly for CAD-CAM milled materials with monomer application (*p* < 0.0001). SB surface treatment for all materials significantly increased SBS when compared with the control and MMA application (*p* < 0.0001).

With monomer application, the CAD-CAM milled groups showed a significant increase in SBS when compared with the HP and 3D-printed groups (*p* < 0.0001). By contrast, the 3D-printed groups showed a significant decrease in SBS compared to HP (*p* < 0.0001). In between the CAD-CAM milled groups, no significant differences were found, and no significant difference between the 3D-printed groups (*p* > 0.05) as well; the highest SBS value was recorded with IvoCad (12.19 ± 1.39 MPa) while the lowest SBS value was recorded with NextDent (2.23 ± 0.35 MPa).

With SB treatment, the CAD-CAM milled groups showed a significant increase in SBS when compared with the HP and 3D-printed groups (*p* < 0.0001). By contrast, the 3D-printed groups showed a significant decrease in SBS compared to HP (*p* < 0.0001). In between CAD-CAM milled groups, significant differences were found, and no significant difference between were found 3D-printed groups (*p* > 0.05). The highest SBS value was recorded with AvaDent (16.64 ± 1.58 MPa) while the lowest SBS value was recorded with ASIGA (5.81 ± 0.88 MPa).

The combined effect of materials and surface treatment on the tested property was analyzed by using two-way ANOVA. The results show that the combined effect of both factors was statistically significant (*p* < 0.0001) (Table 3).

### Nature of Failure

In this study, the nature of the fracture was analyzed in terms of adhesive, cohesive, or mixed as described in previous studies [15,22,23]. Regarding the nature of failure, adhesive failure was dominant in the case of no treatment and made up 100% of failures with ASIGA and NextDent and 90% with FormLabs. The other tested groups showed a less number of cohesive failure, while by the most frequent failure was mixed failure after surface treatment, especially with the SB treatment showing the highest percentage (Table 4).

SEM results of fractured specimens (IvoCad, NextDent, FormLabs, AvaDent, and ASIGA), denture base resins’ sides and repair resins’ sides are displayed in Figure 3. Failure behavior was analyzed in terms of adhesive, cohesive, or mixed. It was observed that IvoCad and NextDent specimens indicated mixed failure (Figure 3A,B) as marked with yellow arrows. The other specimens (FormLabs, AvaDent, and ASIGA) showed the cohesive type of failure, as shown in Figure 3C–E.

## 4. Discussion

In this in vitro study, the effects of the surface treatment on the repair bond strength of CAD-CAM, as well as the conventionally fabricated denture base resins, were tested. Denture base resin types and surface treatment showed significant differences and showed superior bond strength compared with untreated specimens; therefore, the null hypothesis was rejected.

Several attempts have been made to improve the repair strength of the repaired denture base including repair surface design, repair gap modifications, and repair resins reinforcement [16,24,25,26]. Despite the improvement in repair strength with either one application or a combination of these methods, a weak bond at the resin/repair interface was reported in term of adhesive failure [14,15]. To obtain optimum repair strength, the bond between the denture base and the repair martial interface is an essential factor [15,16]. Good bonding depends on different variables such as the original resin and repair resin types, and repair surface treatment (chemical or mechanical method) [14,15]. Surface treatment results in macro and micro irregularities and pits’ formation and increases the surface area at the repair interface [17], in addition to the dissolving of the repair interface followed by diffusion and polymerization of monomers across the repaired interface to form an interpenetrating polymer network [17,21,27]. All of these changes collectively increase the effective bonding area for the micromechanical bonding and retention between the repaired surface and repaired resin [17,18,20].

To evaluate the repair strength of the repaired resins, the flexural strength test has been commonly used in previous studies [24,25,26]. Although it mostly mimics the stress that dentures are subjected to in the oral cavity, SBS was recommended for the evaluation of bond strength at the resin–repair interface [15,18,20,27,28,29]. Based on the objective of this study—examining the bonding strength between repair resins and investigating different denture base resins after surface treatment—the SBS test was selected to compare between the materials. Moreover, the thermal effect was added to the study design, simulating the oral temperature changes, which mainly affect the bond strength [20]. This was attributed to fatigue phenomena, as repeated expansion and shrinkage of the polymer network adversely affects the bond strength through bond rapture [20,30,31]. Furthermore, during thermocycling, the absorbed water leads to the hydrolysis of polymer surfaces [20,32]. Therefore, all repaired specimens were subjected to thermocycling for 5000 cycles, simulating six months of clinical use [33].

In terms of denture base materials, different compositions and different fabrication techniques and technologies affect the properties of final prostheses [7,34]. Two studies primarily investigated the repairability of 3D-printed resin [20] and another study assessed the flexural strength of repaired 3D-printed resin with different surface treatment effects [35]. Promising findings arose regarding the repairability of 3D-printed resin [20] and repaired strength [35], where both studies stated that “3D-printed resin could be repaired and used alternative to conventional denture base resins in terms of repair strength”. However, Li et al. [20] and Neshandar et al. [34], did not compare the 3D-printed resin repair with other different resins repairs. Therefore, the conventional heat-polymerized, CAD-CAM milled, and 3D-printed resins were investigated in the present study, confirming the comparison between different categories.

Conventional as well as CAD-CAM milled (IvoCad, AvaDent) resins showed close SBS, while 3D-printed resins (ASIGA, NextDent, FormLabs) showed a significant decrease in SBS. This may be attributed to the composition and fabrication techniques. Conventional and CAD-CAM milled resins are PMMA-based polymers that are fabricated through heat polymerization in the conventional case and polymerized under high temperature and pressure in the CAD-CAM milled case [36]. This fabrication of specimens from pre-polymerized acrylic resin discs resulted in dentures with high physical and mechanical performances [37], while 3D-printed, ester-based polymers, photo polymerization, and additive manufacturing (layer-by-layer technology) [33] resulted in printed resins with a low rate of double bond conversion [37,38]. This could affect the rapier bond strength as the number of C=C bonds decreases [39].

In the current study, the application of MMA and SB surface treatment significantly increased the shear bond strength of conventionally repaired as well as CAD-CAM fabricated denture base resins. This finding was confirmed based on SEM analysis of the nature of failure, which showed dominance of the cohesive and mixed failure types instead of adhesive failure, which was displayed in no treatment specimens. This is attributed to the aforementioned mechanism of the surface treatment effect, including an increased surface bonding area and micromechanical retention. This is in agreement with previous studies [14,15,21] that showed increased SBS with MMA application [15,18,40] and SB roughening [14,15]. When comparing MMA and SB regarding SBS, SB significantly increased, which is consistent with Qaw et al. [15], who found that SB showed higher SBS compared to monomer application [15]. The SB surface treatments, due to more surface morphologic change, enhances the retention between the denture surface and the repaired materials [15,35].

3D-printed resins showed different behavior in terms of surface treatments, whereas MMA showed no significance with the untreated specimens. However, SB treatment showed a significant increase compared with the no treatment and MMA applications. Evaluation of the nature of failure showed that the dominant fracture type in 3D-printed specimens treated with SB was cohesive. This finding proves the enhancing effect of surface treatment on the bond strength with SB application. 3D-printed resins (ASIGA, NextDent, FormLabs) are light curing materials with bifunctional monomers [41]. Palitsch et al. [41] studied the bond strength of two light curing denture base materials using different conditioning liquids and found that MMA may not be suitable for the bonding with light curing denture base materials, as MMA does not copolymerize with the bifunctional monomer of light curing denture base materials. Additionally, similar findings to Palitsch et al.’s [41] have been reported by previous studies [42,43].

Under all conditions, 3D-printed resins have shown the lowest SBS values. However, comparing these results with previous studies was difficult due to different study designs. In previous studies [18,20,44,45,46], the mean SBS showed high variations (2.0–20 MPa) as reported by Sarac et al. [18] (9.4–20.6 MPa), LI et al. [20] (13.6–17.3 MPa), Ng et al. [44] (14.0–14.8 MPa), Memarian et al. [45] (2.0–9.2 MPa), and Stipho et al. [46] (2.9–5.4 MPa). Compared with the SBS value range reported, the 3D-printed groups with treatment in this study (2.23–6.46 MPa) were in line with the results of Memarian et al. [45] and Stipho et al. [46]. This could confirm the repairability of 3D-printed resins with surface treatments; however, the SBS is still lower than the higher reported values.

The low mechanical performance of 3D-printed resins [33,36] makes them more liable to fracture during service. Accordingly, more attention should be given to 3D-printed denture repair in the future. Based on the finding of this study, the material type affects the repair bond strength, so in case of a CAD-CAM milled denture base being used, surface treatments with MMA and SB are recommended. By contrast, in the case of 3D-printed resins, only SB is recommended for surface treatment. Despite the strength point of the present study (different denture base resins with aging), the results should be taken with caution till further investigation on 3D-printed resins proves these findings.

Among the study limitations, this was an in vitro study where the testing did not completely mimic the clinical conditions; for instance, there was an absence of different oral conditions, repetitive stresses, and masticatory forces as well as different salivary pH effects [47]. Additionally, specimens did not simulate the denture configurations. Although different materials were used, only two surface treatments were evaluated. Therefore, additional investigations under closely simulated clinical conditions are required for evaluating the repair bond strength of CAD-CAM milled and 3D-printed resins using different surface treatments for determining the most appropriate surface treatment.

## 5. Conclusions

Based on the study finding, it can be concluded that:

1. Heat-polymerized and CAD-CAM milled resin showed high repair bond strength compared with 3D-printed resins.
2. MMA and SB surface treatments increased repair bond strength between repair resins and conventional and CAD-CAM milled denture base resins.
3. SB surface treatment of 3D-printed resins increased the SBS between repair resins and 3D-printed resins.
4. There was a positive effect of the SB surface treatment on the investigated resins, making it a suitable surface treatment method for clinical application.

## Figures and Tables

**Table 1:** Summary of materials used in present study and fabrication methods.

Materials	Brand Name	Composition	Specimens’ Fabrication Method
Heat-polymerized acrylic resin (HP)	Major Base.20 (Major Prodotti Dentari Spa, Momcalieri, Italy)	Powder: Polymer (PMMA) þ initiator (benzoyl peroxide [BPO]) (0.5%) þ pigments (salts of cadmium or iron or organic dyes) Liquid: Monomer (MMA) þ cross-linking agent (Ethylene glycol dimethacrylate (EGDMA) 10%) þ inhibitor (hydroquinone)	Conventional heat polymerization method. Polymerization cycle: 90 min in a water bath by heating to 74 °C, then 100 °C for 30 min
CAD/CAM Milled (IvoCad)	IvoCad (Ivoclar Vivadent, Schaan, Liechtenstein)	Prepolymerized PMMA discs 50–100% methyl methacrylate 2.5–10% 1,4-butanediol dimethacrylate	Cut from pre-polymerized acrylic disc using diamond saw (Isomet 5000 Linear Precision Saw, Buehler Ltd., Bluff, IL, USA)
CAD/CAM Milled (AvaDent)	AvaDent (AvaDent Digital Dental Solutions, Scottsdale, AZ, USA)	Prepolymerized PMMA (PMMA 99.5%, pigments < 1.0%)	
3D-printed (ASIGA)	DentaBASE (ASIGA, Erfurt, Germany)	Ethoxylated bisphenol A dimethacrylate 7,7,9 (or 7,9,9)-trimethyl-4,13-dioxo-3,14-dioxa-5,12-diazahexadecane-1,16-diyl bismethacrylate 2- hydroxyethyl methacrylate silicon dioxide diphenyl (2,4,6-trimethylbenzoyl) phosphine oxide titanium dioxide	3D-printed specimens Technology: Printer: ASIGA MAX™ Printing layer thickness: 50 µm Printing orientation: 0-degree Post-curing machine: Asiga-Flash Post-curing time/temp.: 20 min/60 °C
3D-printed (NextDent)	Denture 3D+ NextDent B.V., Soesterberg, The Netherlands	Ester-based monomer; Bisacylphosphine oxide (BAPO) phenylbis (2, 4, 6- trimethylbenzoyl)-phosphine oxide (Omnirad 819)	3D-printed specimens Technology: Printer: NextDent 5100 Printing layer thickness: 50 µm Printing orientation: 0-degree Post-curing machine: LC-D Print Box Post-curing time/temp.: 30 min/60 °C
3D-printed (Formlabs)	Denture Base Resin LP Formlabs Inc., Somerville, MA, USA	55–75% *w*/*w* urethane dimethacrylate, 15–25% *w*/*w* methacrylate monomers, and <0.9% *w*/*w* phenyl bis(2,4,6-trimethylbenzoyl)-phosphine oxide	3D-printed specimens Technology: Printer: Form 2 Printing layer thickness: 50 µm Printing orientation: 0-degree Post-curing machine: FormCure Post-curing time/temp.: 30 min/60 °C

**Table 2:** Mean values, SD, and significance in SBS (MPa) of the experiment groups.

Materials	Surface Treatments			
	Control	MMA	SB	*p*-Value
HP	6.67 ± 1.13 ^a^	10.67 ± 0.87	14.54 ± 1.15	0.000 ^*^
IVOCad	6.87 ± 0.89 ^a^	12.19 ± 1.39 ^a^	16.14 ± 0.95 ^a^	0.000 ^*^
AvaDent	7.08 ± 1.04 ^a^	11.84 ± 1.05 ^a^	16.64 ± 1.58 ^a^	0.000 ^*^
ASIGA	2.77 ± 0.35 ^b,A^	3.47 ± 0.99 ^b,A^	5.81 ± 0.88 ^b^	0.000 ^*^
NextDent	1.91 ± 0.23 ^b,A^	2.23 ± 0.35 ^b,A^	5.85 ± 0.97 ^b^	0.000 ^*^
FormLabs	2.26 ± 0.32 ^b,A^	3.81 ± 0.72 ^b,A^	6.46 ± 0.92 ^b^	0.000 ^*^
*p*-value	0.000 ^*^	0.000 ^*^	0.000 ^*^	

* Statistically significant *p* < 0.05 level of significance; same small letters vertically in each column indicate a non-significant difference between the pairs, while same capital letters in each row indicate a non-significant difference between the pairs horizontally.

**Table 3:** Two-way ANOVA results showing combined effect of materials and surface treatment.

Source	Type III Sum of Squares	df	Mean Square	F-Test	*p*-Value
Material	2608.361	5	521.672	579.472	0.000 *
Surface treatment	1201.135	2	600.567	667.109	0.000 *
Material * Surface treatment	233.158	10	23.316	25.899	0.000 *
Error	145.841	162	0.900		
Total	14,658.535	180			

* Statistically significant at a *p* < 0.05 level of significance.

**Table 4:** Nature of failure in numbers and percentages.

Martials	Surface Treatment	Nature of Failure %		
		Adhesive	Cohesive	Mixed
HP	Control	7(70)	--	3(30)
	MMA	4(40)	2(20)	4(40)
	SB	3(30)	--	7(70)
IvoCad	Control	6(60)	1(10)	3(30)
	MMA	3(30)	1(10)	6(60)
	SB	---	3(30)	7(70)
AvaDent	Control	4(40)	1(10)	5(50)
	MMA	2(20)	2(20)	6(60)
	SB	--	4(40)	6(60)
ASIGA	Control	10(100)	--	--
	MMA	8(80)	1(10)	1(10)
	SB	3(30)	5(50)	2(20)
NextDent	Control	10(100)	--	--
	MMA	7(70)	1(10)	2(20)
	SB	2(20)	5(5)	3(30)
FormLabs	Control	9(90)	--	1(10)
	MMA	6(60)	-	4(40)
	SB	3(30)	5(50)	2(20)

MMA: methyl meth acrylate; SB: alumina blasting.

## Data Availability

Not applicable.
